# Magnetic Frequency Tuning of a Multimodal Vibration Energy Harvester †

**DOI:** 10.3390/s19051149

**Published:** 2019-03-07

**Authors:** Sofiane Bouhedma, Yuhang Zheng, Fred Lange, Dennis Hohlfeld

**Affiliations:** Institute for Electronic Appliances and Circuits, Faculty of Computer Science and Electrical Engineering, University of Rostock, Albert-Einstein-Str. 2, 18059 Rostock, Germany; yuhang.zheng@uni-rostock.de (Y.Z.); fred.lange@uni-rostock.de (F.L.); dennis.hohlfeld@uni-rostock.de (D.H.)

**Keywords:** energy harvesting, vibration, piezoelectricity, nonlinear resonators, magnetic frequency tuning, multimodal structures, bi-stability

## Abstract

In this paper, we present a novel vibration-based piezoelectric energy harvester, capable of collecting power at multiple operating frequencies and autonomously adapting itself to the dominant ambient frequencies. It consists of a compact dual-frequency resonator designed such that the first two fundamental natural frequencies are in the range of [50, 100] Hz, which is a typical frequency range for ambient vibrations in industrial environments. A magnetic frequency-tuning scheme is incorporated into the structure, which enables the frequency agility of the system. In contrast to single frequency harvesters, the presented approach combines multi-resonance and frequency tunability of both modes enabling a larger operative bandwidth. We experimentally demonstrate independent bi-directional tunability of our dual-frequency design. Furthermore, a control algorithm based on maximum amplitude tracking has been implemented for self-adaption of the system. The latter has been demonstrated in a system-level simulation model, which integrates the dual-frequency resonator, the magnetic tuning, and the control algorithm.

## 1. Introduction

The increasing demand for smart condition monitoring systems in industrial or automotive applications calls for deployment of wireless sensor networks in inaccessible or harsh environments, in which battery use is not possible and where energy harvesting systems represent a better candidate to act as a local sustainable power source. The ‘first generation’ vibration-based energy harvesting devices consist mainly of spring-mass-damper systems, generating maximum power, only when their natural frequencies coincide exactly with the dominant ambient frequency. Therefore, they can be applied only if the vibration frequency is known beforehand. Realistic ambient vibration spectra exhibit multiple frequencies, which also shift over time as the vibration source is aging or changing in temperature. These resonant energy harvesting schemes fail in such environments. Hence, frequency-agile systems (i.e., capable of adjusting their operation frequency) and multimodal-based harvesters have been proposed to overcome this challenge.

In recent years, numerous multimodal resonator designs have been introduced. Shaofan et al. [1] proposed a multi-resonant structure comprising a clamped–clamped piezoelectric fiber composite generator with side mounted cantilevers, which are tuned by added masses to be resonant at different frequencies, inducing a wider harvesting bandwidth. A novel piezoelectric vibration energy harvester, consisting of a stack of five resonators has been proposed in [2] for harvesting power at multiple frequencies. The work presented in [3] investigates a compact piezoelectric energy harvester, comprising one main cantilever beam and an inner secondary cantilever beam and efficiently harvests power at two distinct frequencies. A novel trident (three-pronged spear) shaped piezoelectric energy harvester has been proposed in [4] to collect power from wideband, low-frequency, and low-amplitude ambient vibrations. Ramírez et al. [5] analyzed the effects of rotational motion on the performance of a novel piezoelectric energy harvester, consisting of a two E-shape multiple beams system joined with a rigid beam and operating at low frequencies. The drawback of this approach is the limited operational bandwidth, achievable only by increasing the overall size of the harvester, which on the contrary, needs to be minimized in most industrial applications.

Alternatively, various groups [6,7,8,9,10] demonstrated the usability of bi-stable resonators for harvesting on a wider bandwidth, by integrating permanent magnets positioned with respect to another permanent magnet on the resonator. In [11,12] a tuning mechanism has been developed, allowing compensation of the hysteresis as well as maintaining the optimal working point. Other research [13] adapts the magnetic field strength, acting on the resonator, by orienting a circular permanent magnet. The mechanism allows the use of both coupling modes (attractive and repulsive), which enables the harvester to adapt its operating frequency to the dominant vibration frequency of the environment. The harvester itself ensures the provision of the required power for frequency tuning. In [14] an enhanced novel design of a nonlinear magnetic levitation-based energy harvester has been proposed, where the tuning effect is achieved by the magnetic and the oblique springs. A bi-stable rotational energy harvester with wide bandwidth and operating at low frequencies has been proposed in [15,16]. The energy conversion is achieved by magnetic plucking of a piezoelectric cantilever using a driving magnet mounted on a rotating platform. In [17] a compact nonlinear multi-stable energy harvester array has been presented for harvesting energy at low frequencies. In [18,19,20] we studied the behavior of a dual-frequency piezoelectric energy harvester incorporating permanent magnets for bi-directional frequency tuning. Other groups used different approaches for broadening the harvesting bandwidth, for example, Ref. [21] analyzed a broadband energy harvester with an array of piezoelectric bimorphs mechanically connected via springs. The operative bandwidth broadening can be achieved by carefully selecting the masses and adjusting the spring stiffness. Adaptive power harvesters using shunted piezoelectric control system have been proposed in [22,23], providing effective broadband power generation for application in wireless sensor devices.

**Figure 1:**
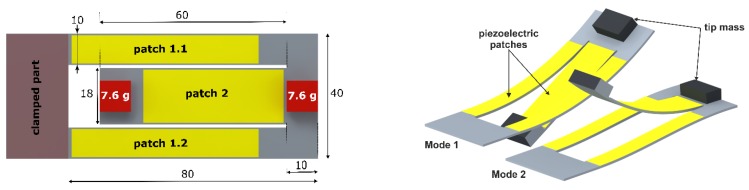
Geometry description (dimensions in mm) of the dual-frequency piezoelectric energy harvester (**left**), first two fundamental mode shapes (**right**) appearing at two close frequencies.

Despite the considerable amount of research and the numerous attempts which have been made using various approaches, achieving multiple resonant peaks in a specified frequency range to facilitate broadband energy harvesting remains a challenging task. In this paper, we characterize the dual-frequency piezoelectric energy harvester. The geometry consists of a so-called folded beam resonator (fabricated from a single steel sheet), integrating permanent magnets at the free ends of the cantilevers. The design has been developed such that two fundamental modes, corresponding respectively to the outer and inner beam, exist at distinct frequencies (Figure 1) in the frequency domain [50, 100] Hz. In contrast to [3], a bi-directional frequency tuning was achieved by symmetrically arranging external magnets and adjusting their positions. It is a unique feature of our approach that both modes can be tuned independently. The dual frequency feature of the structure, together with the independent bidirectional frequency tuning of each mode, provide superior frequency agility and increase the operational bandwidth of the system compared to existing approaches. A control scheme, which uses an energy efficient maximum-amplitude-tracking algorithm, is simulated together with a lumped model of a frequency tunable single- and dual frequency resonator. The position-dependent magnetic force has been obtained from magnetostatic simulations. The system is able to autonomously choose and tune the closest mode to the dominant vibration frequency to maintain maximum possible oscillation amplitude. Furthermore, we propose a unique reluctance-based tuning scheme, incorporating a single diametrically polarized magnet and two permalloy yokes with very high permeability. The angular positioning of the magnet will replace the linear actuation of the two pairs of magnets in the actual tuning mechanism, which will enhance the power budget of the whole system. The simulations demonstrated that a better tuning efficiency can be achieved compared to the standard tuning mechanisms, requiring linear magnets actuation.

## 2. Dual Frequency Piezoelectric Energy Harvester

In order to simulate the behavior and estimate the power output of the proposed energy harvester, we implemented the geometry in the finite element-based simulation tool ANSYS Multiphysics, as depicted in Figure 1. The mechanical resonator consists of a folded beam structure, mainly two identical 80 mm long arms (referred to as outer beams), stretching from the base and mechanically connected via a common end to a 60 mm long inner beam, which extends towards the base. We considered the mechanical resonator with two identical masses *m* = 7.6 g. The modal analysis yielded the mode shapes at two natural frequencies. The first two resonance frequencies obtained from the simulation, which are *f*_1,*sim*_ = 63.182 Hz and *f*_2,*sim*_ = 77.457 Hz, respectively have been compared to the corresponding experimental values and show a very good agreement. In particular, we measured *f*_1,*exp*_ = 62.630 Hz and *f*_2,*exp*_ = 76.072 Hz. The model has been validated through further comparisons between the harmonic analysis and the experimental results [19].

**Figure 2:**
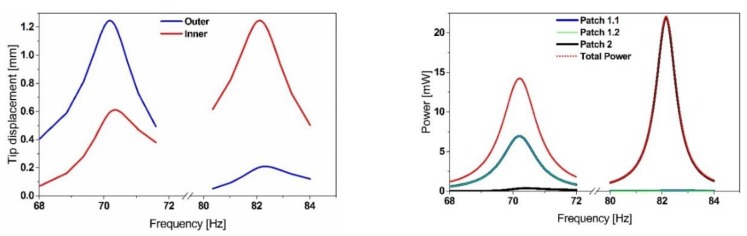
Simulated power output of the proposed energy harvester, illustrating the dual frequency operation of the structure with comparable power output levels.

We furthermore implemented piezoelectric films, (60 × 10 × 0.2 mm^3^ on the outer beam and 48 × 18 × 0.2 mm^3^ for the inner beam), into our ANSYS model. Patch 1.1 and 1.2 are electrically connected in parallel, whereas patch 2 is operated independently since it operates at a different frequency and thus requires a different optimum load. Through a harmonic analysis, we estimated the voltage output. The power output is then given by the following expression [24]:

(1) $$P_{ij}=\frac{1}{4}V_{0j}^{2}2\pi f_{j}C_{i}$$

where: *V*_0_*_j_* is the open-circuit output voltage amplitude at mode *j*, *f_j_* is the mode frequency, and *C_i_* is the capacitance of the piezoelectric patch *i*. The total power output represented in Figure 2, is then the sum of all power contributions delivered by each patch at every mode. The material properties of the piezoelectric ceramic correspond to the material PIC-255, supplied by PI Ceramic and are given in Table A1 in the Appendix A.

The previous results (Figure 2) show that the considered resonator amplifies the displacement at the first and the second mode significantly, leading to a distribution of the mechanical strain over the flexible structure. Consequently, the corresponding piezoelectric film polarizes and generates surface charges (voltage).

## 3. Frequency Tuning

**Figure 3:**
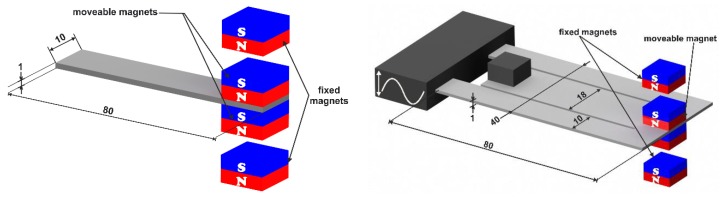
Geometry description of the nonlinear simple cantilever resonator (**left**) and the dual frequency design (**right**).

In this paper, we propose magnetic frequency tuning. The approach consists of integrating permanent magnets with the resonating structure together with fixed magnets. They can be incorporated in different configurations, for instance in an axial configuration, such that the generated force reactions will be coplanar with the structure axis, or alternatively, vertical to the structure’s surface, which will be considered in this paper and referred to as a vertical configuration (Figure 3). The interactions between the magnets create an effective spring constant. The latter will sum-up the mechanical stiffness of the structure and lead to hardening or softening, depending on the ‘repelling’ or ‘attractive’ orientation of the magnets. This leads to a frequency up or down tuning, respectively.

### 3.1. Bi-Directional Frequency Tuning Simulation

**Figure 4:**
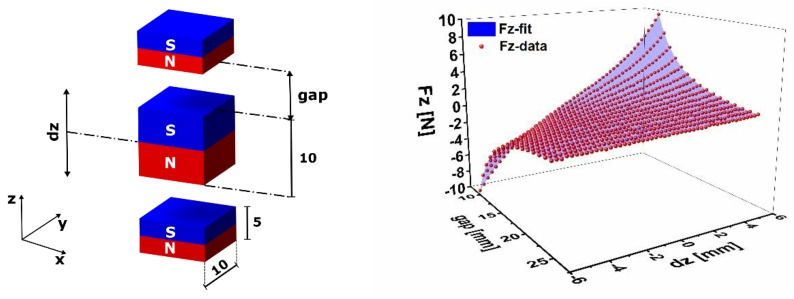
Magnet configuration considered in the magnetostatic simulation (**left**), together with the force-gap-displacement function for the attractive force (**right**).

The simulation of the bi-directional frequency tuning effect in different configurations and orientations requires the implementation of the magnetic forces. Therefore, we considered a parametrized magnetostatic simulation of a configuration of neodymium permanent magnets with N42 magnetization as shown in Figure 4.

The dimensions of the outer magnets are 10 × 10 × 5 mm^3^, whereas the inner magnet has an identical cross-section and a thickness of 10 mm. For simplification purposes, we neglect the rotation of the magnet as the beam undergoes deflection and consider only vertical displacements. Here, we consider attracting magnets in the vertical configuration. The simulation has been performed for different gap values (reported as the distance between the fixed and the movable magnet at the rest position) in the range 10 to 25 mm and for different vertical displacements of the movable magnet in the interval −6 to 6 mm. In order to include the effect of the magnetic tuning, we consider spring elements with displacement-dependent, hence nonlinear, stiffness. Therefore, we derived a two-variable fitting function (Equation (2)).

(2) $$F=\overset{7}{\underset{\begin{array}{c} i=0\\ j=7-i \end{array}}{\sum }}a_{ij}x^{i}z^{j}$$

where *x* and *z* are the gap and the vertical displacement, respectively. The constants *a_ij_*, are given in Table A2 in the Appendix A. The fitting function shows an excellent match with the simulation results (Figure 4). As a first step, the defined functions have been implemented into a two-dimensional ANSYS model of the simple cantilever resonator in Figure 3 (left). The magnetostatic forces are represented by the nonlinear spring element COMBIN39.

**Figure 5:**
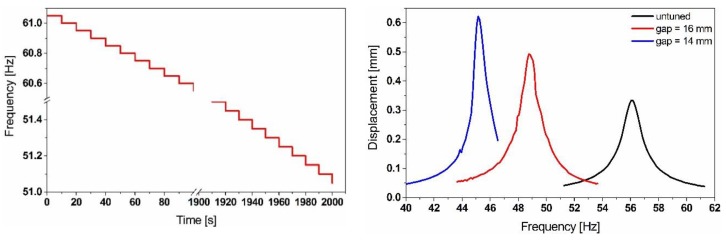
Transient analysis simulation results of the simple cantilever resonator full model for several gap values, undergoing a stepwise frequency-varying base displacement (**left**) and showing the frequency shift of the initial resonance (**right**).

The untuned natural frequency of the resonator is *f*_0_ = 56.296 Hz. By considering attractive forces, the overall stiffness of the structure will decrease. We run a transient simulation with a harmonic base excitation and a stepwise decreasing excitation frequency (sweep down ‘s.d.’) as shown in Figure 5. Each frequency step needs to run for 10 s, in order to make sure that the system reaches the steady-state regime at which oscillation amplitude is obtained.

**Figure 6:**
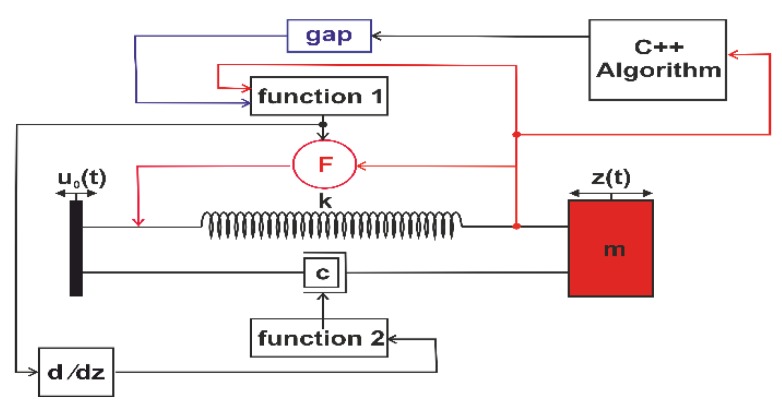
Descriptive scheme of the nonlinear simple-cantilever resonator geometry and its equivalent compact model.

The graph shown in Figure 5 represents transient analysis results. They demonstrate the frequency tuning effect of the initial resonance of the resonator, showing that the magnetic forces implemented into our model lead to softening and the results match with the experimental ones. Due to the considerable solution time of a full transient simulation, we considered an equivalent nonlinear compact model, consisting of a lumped spring-mass-damper resonator in ANSYS Twin Builder (see Figure 6). We parametrized the excitation frequency such that it sweeps in both directions. The magnetic force has been implemented as a two-variable-force function denoted as function 1. While function 2, needed to calculate the variable damping coefficient, is the overall stiffness of the system. It includes the spring constant and the magnetic stiffness (derivative of the force expression with respect to the vertical displacement) arising from the magnetic interaction between the moving and the fixed magnets as described in Equation (8).

The equation of motion of a standard linear single degree of freedom system (SDOF), undergoing a harmonic base excitation $u_{0}\left(t\right)$ can be expressed in Equation (3).

(3) $$m\ddot{z}+c\dot{z}+kz=c{\dot{u}}_{0}+ku_{0}$$

where *m*, *c*, and *k* are the equivalent mass, the damping coefficient and the spring constant, respectively. According to [25], the equivalent mass of tip-loaded uniform cantilever can be calculated using Equation (4).

(4) $$m=m_{t}+\frac{33}{140}m_{b}$$

where *m_t_* is the tip mass, which represents in our case the moveable magnet’s weight and *m_b_* is the beam mass. If *E*, *I* and *l* are the Young’s modulus, the moment of inertia and the length of the simple cantilever resonator, the beam’s stiffness *k* is given by Equation (5).

(5) $$k=\frac{3EI}{l^{3}}$$

In the following, we consider a constant damping ratio of ξ=0.015, which enables us to define the damping constant *c*, which can be calculated then using Equation (6).

(6) $$c=2\xi \sqrt{mk}$$

However, for the nonlinear SDOF resonators, the stiffness will depend on displacement as described in Equation (7).

(7) $$m\ddot{z}+c\dot{z}+k_{t}\left(z\right)z=c{\dot{u}}_{0}+k_{t}u_{0}$$

where *k_t_* denotes the overall stiffness of the system, which is the superposition of the stiffness generated by the permanent magnets and the equivalent stiffness of cantilever. The first partial derivative of the force expression (Equation (2)) with respect to the displacement *z* will define an effective magnetic stiffness.

(8) $$k_{t}\left(z\right)=k\pm k_{m}\left(z\right)\;k_{m}\left(z\right)=\frac{\partial F}{\partial z}dz$$

**Figure 7:**
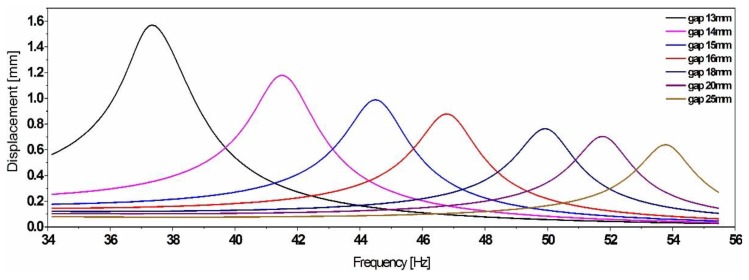
Simulation results of the equivalent compact model, illustrating the frequency shift and the amplitude variation while down tuning.

The frequency down-tuning results of the equivalent compact model represented in Figure 7 show a good match in terms of the frequency with the full model.

### 3.2. Experimental Work

**Figure 8:**
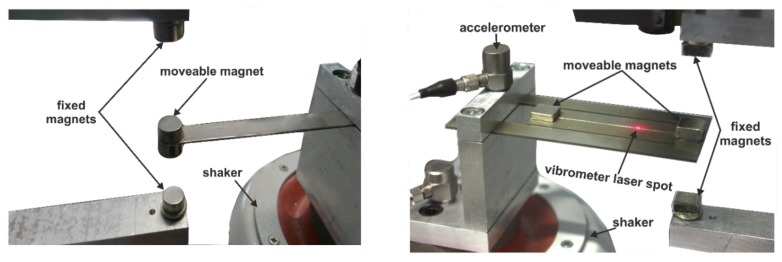
Experimental test bench used for the frequency tuning investigations of a simple cantilever resonator (**left**) and the folded beam structure (**right**).

In order to experimentally investigate the magnetic frequency tuning capabilities, we first considered a simple cantilever resonator (stainless steel, 80 × 10 × 1 mm^3^, see Figure 3). The structure is excited with a harmonic base acceleration of *a* = 0.2 g generated by test equipment (Figure 8).

The tuning mechanism can incorporate permanent Neodymium (NdFeB) magnets in attractive and repulsive orientations, assembled in a vertical configuration. The positions of the magnets are adjusted using computer-controlled motorized linear stages.

**Figure 9:**
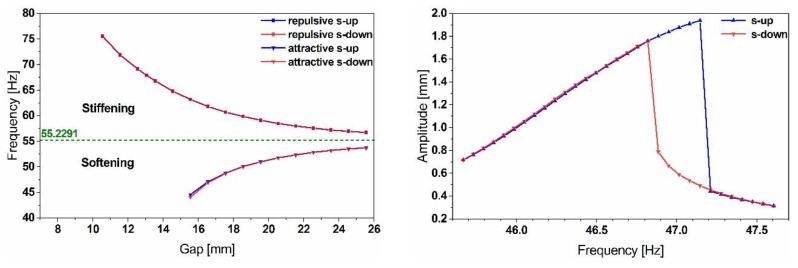
Experimental results showing the bidirectional frequency tuning of a simple cantilever resonator, by up to 35% (**left**) and illustrating the hysteresis effect observed while tuning at a gap value of 10 mm (**right**) and caused by the strong nonlinearity of the magnetic forces.

The results in Figure 9 illustrate the bidirectional tuning of the simple cantilever resonator’s frequency by up to 35% out of a center frequency. Nevertheless, the system becomes highly unstable, while tuning at smaller gap values, which translates into the hysteresis effect depicted in Figure 9 and illustrated by the resonance shift between the frequency sweep up (s-up) and sweep down (s-down).

**Figure 10:**
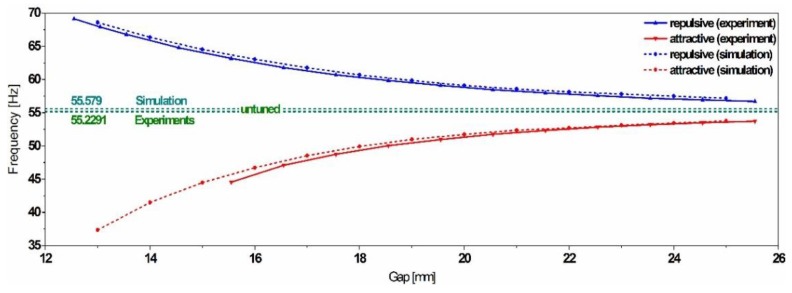
Experimental validation of the compact model and the magnetic forces implementation.

The experimental results validate the compact model and demonstrate the reliability of the magnetic forces implementation. The comparison shows a minor deviation between the simulation and the experimental data, with an estimated error below 1%, as illustrated in Figure 10.

The experiments have been extended to consider the dual-frequency stainless steel resonator. This time, the second pair of NdFeB magnets has been integrated to act on the inner beam.

**Figure 11:**
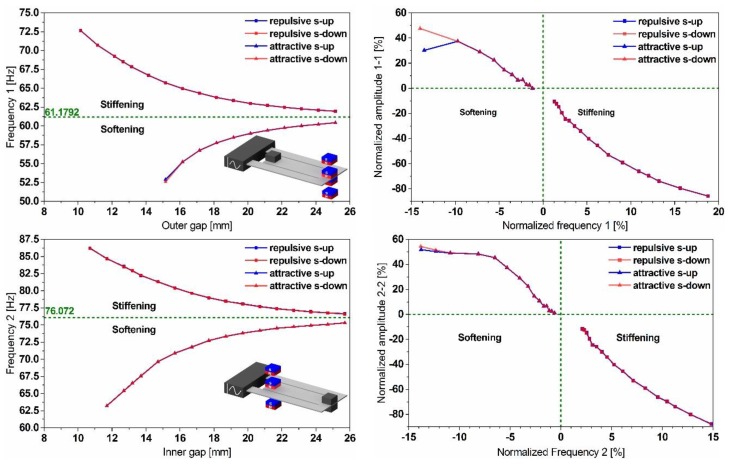
Experimental results of the bidirectional magnetic frequency tuning of the dual-frequency resonator, demonstrating frequency agility varying between −14% and +18% (**left**). Moreover, they illustrate the amplitude variation accompanying frequency tuning (**right**).

**Figure 12:**
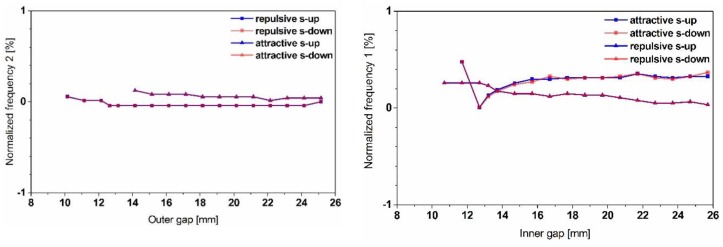
Experimental dataset demonstrating the independence between tuning mode 1 (**left**), respectively mode 2 (**right**).

The results in Figure 11 show the effect of the gap variation on both modes’ frequencies of the dual-frequency resonator and illustrate a maximum bidirectional frequency shift of up to 18%. However, the decrease in the displacement amplitudes, observed while up tuning, constitutes a limitation of such a strategy. The independence of tuning both frequencies shown in Figure 12, opens up the opportunity to reduce the frequency gap between the two modes and enables the possibility for a frequency overlap.

### 3.3. Frequency Tuning Control Scheme

**Figure 13:**
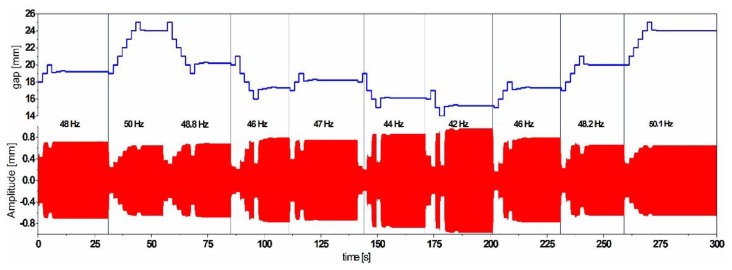
Simulation results of the implemented frequency tuning control scheme, tracking the maximum amplitude through multiple frequency states.

Based on the tuning results, a control scheme, which uses an energy efficient maximum-amplitude-tracking algorithm, has been developed and simulated for a frequency tunable simple resonator. The algorithm is able to self-adapt the gap step size to maintain maximum possible oscillation amplitude. In order to check the performances of the algorithm, we excited the simple cantilever resonator with a step-wise frequency-varying harmonic base acceleration with an amplitude of 0.2 g as depicted in Figure 13.

The results illustrate the self-adaption of the resonator’s natural frequency according to the most dominant excitation frequency. The adaptive gap step size enables getting as close as possible to the resonance to ensure the maximum possible amplitude and increases the power efficiency of the system by reaching the desired frequency with a minimum number of steps.

**Figure 14:**
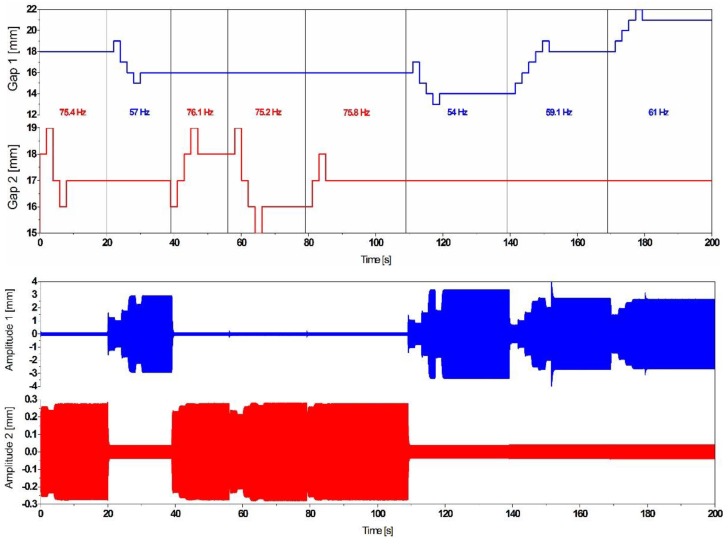
Simulation results of autonomous frequency tuning by a maximum-amplitude-tracking-based algorithm, which identifies the dominant frequency and tunes the closest mode.

Additionally, the implemented algorithm has been extended to consider the dual frequency resonator and improved in such a way that it enables the system to autonomously choose and tune the closest mode to the dominant vibration frequency, as illustrated in Figure 14, by triggering one of the tuning mechanisms and maintaining the second one in a sleep mode.

The response of the resonator under a frequency varying excitation represented in Figure 14 demonstrates the smart self-adaption of the resonator to the closest excitation frequency.

### 3.4. Novel Tuning Approach

**Figure 15:**
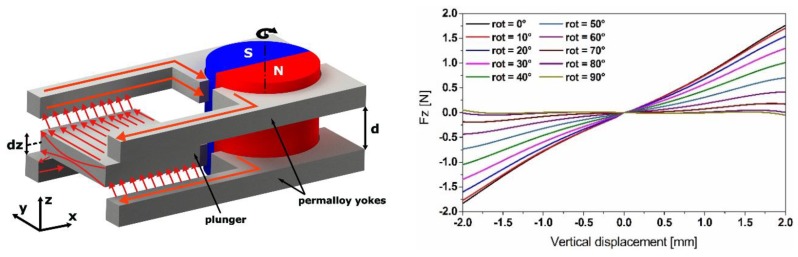
Reluctance-based tuning scheme with low power requirements, where the tuning is achieved by the rotation of the permanent magnet, enabling structure softening. The resonator carries high permeability material as a magnetic plunger (**left**), together with the simulation results of the magnetic forces acting on the resonator (**right**).

The tuning mechanism based on a rotational disc magnet proposed in [13] shows a low power consumption compared to the linear positioning-based tuning mechanisms. In order to ensure the efficiency of our harvesting device, we propose a reluctance-based tuning scheme incorporating a single diametrically polarized magnet with rotational actuation. Tuning is achieved by changing the angular position of the magnet, which replaces the linear positioning of the external magnets in the actual mechanism. A magnetic yoke fabricated from high permeability material (mu-metal) guides the magnetic flux towards another mu-metal component attached to the free end of the cantilever as a tip mass, which will be moving as a plunger, while the resonator starts to oscillate (Figure 15).

**Figure 16:**
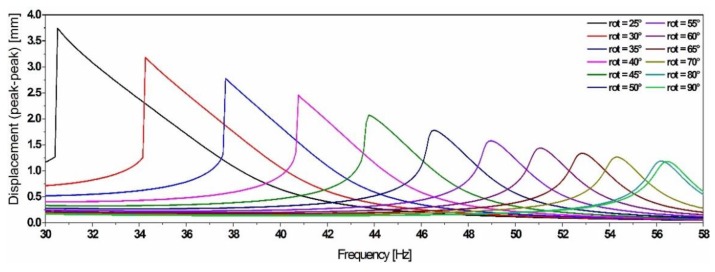
Simulation results of the equivalent compact model, illustrating the frequency shift (up to 45%) and the amplitude variation while down tuning using the reluctance-based tuning scheme.

The tunability has been numerically demonstrated by changing the angular position of the magnet in a magnetostatic simulation. In order to compare the efficiency of the reluctance-based tuning scheme, with the previous tuning scheme based on the linear positioning of the permanent magnets, we used a similar N42 NdFeB magnet, but diametrically polarized, providing a magnetic flux density of *B* = 1.32 T. The material properties of ASTM A 753 provided by ‘Magnetic Shields^®^’ (80%Ni15%Fe%5Mo, *μ_r_* = 470.000, saturation of 0.6 T) has been implemented in the model. The reluctance force (represented in Figure 15) has been implemented in the same equivalent compact model of the simple cantilever resonator as shown in Figure 16.

These results demonstrate that by using the proposed scheme, the frequency tuning efficiency can be improved compared to the magnets-linear-positioning-based tuning scheme. Subsequently, they illustrate that the optimum tuning efficiency can be achieved for angular positions of the magnet in the range between 70° and 25°. For lower angular values, the system becomes bi-stable.

## 4. Conclusions and Outlooks

In this work, we presented a novel self-tunable dual-frequency piezoelectric energy harvester. The dual frequency feature has been demonstrated through simulations, which showed that the resonator magnifies the amplitudes at two close resonances, enabling harvesting power at both modes. The frequency tunability of the system has been demonstrated experimentally by integrating permanent magnets in different orientations, which causes a structure hardening or softening and leads to a frequency up and down tuning, respectively. It has been shown that the frequency can be tuned by up to 18% in both directions and for both modes independently, which increases the frequency agility of our system, compared to other designs. Nevertheless, an amplitude decrease has been observed, while up-tuning the frequency, which limits the use of this approach.

Moreover, in order to simulate the bi-directional frequency tuning of the resonator and define the force interactions between the permanent magnets, a magnetostatic simulation has been performed. The results of this simulation enabled us to define force functions giving a general force-gap-displacement relationship, which has been implemented into a 2D simple resonator model. The simulation results demonstrated that the implemented forces trigger a structure softening leading to a decrease of the natural frequency. Due to the considerable solution time of a full F.E. model, an equivalent compact model of the nonlinear resonator has been developed and validated by experimental work. Beyond establishing and simulating the behavior of the nonlinear resonator, a control scheme, based on an energy efficient maximum-amplitude-tracking algorithm, has been developed. It integrates the possibility to autonomously choose and tune the closest mode to the dominant vibration frequency, with a self-adaptive step size to maintain maximum oscillation amplitude.

Finally, in order to ensure the efficiency of the harvesting device, we propose to improve the tuning scheme used to demonstrate the frequency agility of the resonator. We propose a reluctance-based tuning scheme incorporating a single actuated diametrically polarized magnet. The mechanism has a lower power budget compared to the linear magnets positioning, due to the lower number of actuation needed for the tuning. Through a simulation investigation, we demonstrated that the frequency tuning range can be improved compared to the magnets linear positioning scheme. An extensive experimental investigation to validate the simulation outcomes is planned.

## Appendix A

**Table A1:** Material properties and dimensions of the piezoelectric film PIC-255.

**Mechanical Properties**		**Dimensions (length × width × thickness [mm^3^])**	
Density [kg/m^3^]	7800	Patch 1.1/1.2	60 × 10 × 0.2
Poisson’s ratio	0.36	Patch 2	48 × 18 × 0.2
**Elasticity Coefficients [×10^10^ N/m^2^]**		**Piezoelectric Constants [N/Vm]**	
*C_11E_*	12.30	*e_31_*	−7.150
*C_33E_*	9.711	*e_33_*	13.75
*C_44E_*	2.226	*e_15_*	11.91
*C_66E_*	2.315	**Dielectric Coefficients**	
*C_12E_*	7.670	*ɛ_11_*	930
*C_13E_*	7.025	*ɛ_33_*	857

**Table A2:** Coefficients of the two-variable fit function expressing the magnetic force as a function of the gap and the vertical displacement.

$a_{00}$	1.376 × 10^1^	$a_{03}$	1.839 × 10^−1^	$a_{23}$	2.853 × 10^−3^	$a_{06}$	2.071 × 10^−6^
$a_{10}$	−6.894 × 10^0^	$a_{40}$	1.033 × 10^−2^	$a_{14}$	1.291 × 10^−4^	$a_{70}$	−6.909 × 10^−8^
$a_{01}$	1.651 × 10^1^	$a_{31}$	−3.492 × 10^−2^	$a_{05}$	3.570 × 10^−4^	$a_{61}$	2.117 × 10^−7^
$a_{20}$	1.428 × 10^0^	$a_{22}$	3.846 × 10^−3^	$a_{60}$	8.039 × 10^−6^	$a_{52}$	−7.301 × 10^−8^
$a_{11}$	−4.414 × 10^0^	$a_{13}$	−3.739 × 10^−2^	$a_{51}$	−2.595 × 10^−5^	$a_{43}$	1.198 × 10^−6^
$a_{02}$	1.139 × 10^−1^	$a_{04}$	−8.264 × 10^−4^	$a_{42}$	6.434 × 10^−6^	$a_{34}$	1.148 × 10^−7^
$a_{30}$	−1.591 × 10^−1^	$a_{50}$	−3.916 × 10^−4^	$a_{33}$	−9.604 × 10^−5^	$a_{25}$	7.225 × 10^−7^
$a_{21}$	5.290 × 10^−1^	$a_{41}$	1.307 × 10^−3^	$a_{24}$	−6.801 × 10^−6^	$a_{16}$	−6.097 × 10^−8^
$a_{12}$	−3.301 × 10^−2^	$a_{32}$	−2.234 × 10^−4^	$a_{15}$	−3.120 × 10^−5^	$a_{07}$	−3.415 × 10^−7^
